# Statistical Analysis of the Processes Controlling Choline and Ethanolamine Glycerophospholipid Molecular Species Composition

**DOI:** 10.1371/journal.pone.0037293

**Published:** 2012-05-25

**Authors:** Kourosh Zarringhalam, Lu Zhang, Michael A. Kiebish, Kui Yang, Xianlin Han, Richard W. Gross, Jeffrey Chuang

**Affiliations:** 1 Department of Biology, Boston College, Chestnut Hill, Massachusetts, United States of America; 2 Division of Bioorganic Chemistry and Molecular Pharmacology, Department of Internal Medicine, Washington University School of Medicine, St. Louis, Missouri, United States of America; 3 Sanford Burnham Medical Research Institute, Diabetes and Obesity Research Center, Orlando, Florida, United States of America; University of Cambridge, United Kingdom

## Abstract

The regulation and maintenance of the cellular lipidome through biosynthetic, remodeling, and catabolic mechanisms are critical for biological homeostasis during development, health and disease. These complex mechanisms control the architectures of lipid molecular species, which have diverse yet highly regulated fatty acid chains at both the sn1 and sn2 positions. Phosphatidylcholine (PC) and phosphatidylethanolamine (PE) serve as the predominant biophysical scaffolds in membranes, acting as reservoirs for potent lipid signals and regulating numerous enzymatic processes. Here we report the first rigorous computational dissection of the mechanisms influencing PC and PE molecular architectures from high-throughput shotgun lipidomic data. Using novel statistical approaches, we have analyzed multidimensional mass spectrometry-based shotgun lipidomic data from developmental mouse heart and mature mouse heart, lung, brain, and liver tissues. We show that in PC and PE, sn1 and sn2 positions are largely independent, though for low abundance species regulatory processes may interact with both the sn1 and sn2 chain simultaneously, leading to cooperative effects. Chains with similar biochemical properties appear to be remodeled similarly. We also see that sn2 positions are more regulated than sn1, and that PC exhibits stronger cooperative effects than PE. A key aspect of our work is a novel statistically rigorous approach to determine cooperativity based on a modified Fisher's exact test using Markov Chain Monte Carlo sampling. This computational approach provides a novel tool for developing mechanistic insight into lipidomic regulation.

## Introduction

The cellular lipidome is comprised of diverse classes of sphingolipids, phospholipids, glycerolipids, sterol lipids, and lipid metabolites, whose molecular species coordinate biomembrane structure, intra- and extra-cellular communication, metabolic efficiency, and signaling cascades that are critical for cellular functionality in development and disease [1], [2]. Identification and quantification of thousands of lipid molecular species, including regioisomers, are now possible due to advances in soft ionization mass spectrometry as well as novel chemical strategies [3]–[6]. As with other -omics sciences, lipidomics now requires more advanced integration of computational and statistical approaches to interpret accruing datasets of complex distributions of lipid molecular species, which have broad and potent functional significance [7]. Thus, advances in computational lipidomics can dramatically improve our understanding of the functions of the cellular lipidome.

Glycerophospholipids comprise the vast majority of membrane lipid content. Each is composed of a glycerol backbone, a head group esterified to a phosphate that connects to the glycerol at the sn3 position, and acyl chains located at the sn1 and the sn2 positions of the glycerol [8]. Multidimensional mass spectrometry-based shotgun lipidomics (MDMS-SL) using dimensional, chemical, and computational strategies have shown that lipid molecular species have diverse, highly regulated acyl chains at specific positions [3], [4]. The distribution and content of molecular species are selectively regulated by the complex homeostatic balance of biosynthesis, remodeling (transacylase or acyltransferase), and catabolism [9], [10]. In the vast majority of tissues and membranes, the two most abundant glycerophospholipids are PC and PE. It is roughly known that shorter, saturated acyl chains are localized in the sn1 positions and longer, more unsaturated acyl chains are enriched in the sn2 positions, likely due to biophysical stringency and positional functional recognition by phospholipases. However, such characterizations of PC and PE acyl chains have not been analyzed in any statistical framework, in spite of the fact that high-throughput lipidomic data are now available. Lipidomic data provide an opportunity for rigorous identification of PC and PE acyl chain behaviors, a vital step in determination of mechanisms of acyl chain regulation.

Some computational approaches have been developed for lipidomics, notably for the problems of identifying low abundance lipid molecular species or for dissecting lipid metabolic signaling pathways at the class level [7], [11]–[16]. Processes controlling species composition have been previously investigated for the tetra-acyl phospholipid cardiolipin [17], in the context of a simplified model of independent and identical behavior of the acyl chains. However, the extent of cooperative interactions among acyl chains within a single phospholipid (e.g. cooperation between sn1 and sn2 positions, an idea proposed for cardiolipin by Schlame et al [18]) is poorly understood. Quantification of phospholipid acyl behaviors is vital for understanding the regulation of biochemical functions controlled by phospholipids. Knowledge of these behaviors will also improve detection of molecular species lying just below current limits of lipidomic measurement technology via cryptoanalytical approaches that combine chemical detection with computational simulation of acyl chain remodeling behaviors [15]. Lipid biochemistry will soon rely increasingly on this type of mechanistic strategy to further penetrate and integrate the cellular lipidome.

In this study, we present a computational analysis of the dependence between acyl chains in the phosphatidylcholine and phosphatidylethanolamine molecular species. PC and PE are critical molecules because of their dominance in the phospholipid composition of cellular membranes. Also because these molecules each have only two acyl chains, they are the simplest types of lipids for which to investigate cooperative effects. For this analysis, we have analyzed MDMS-SL measurements of PC and PE in mouse heart development (at days 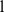, 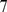, 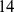, 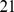, 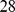, 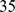, and 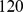) as well as three other mature mouse tissues (brain, lung, liver).

We demonstrate several key findings. We show that in PC and PE sn1 and sn2 positions are largely independent, though for low abundance species there may be processes controlling species composition that involve both the sn1 and sn2 chains simultaneously, leading to cooperative effects. Chains with similar biochemical properties appear to be regulated similarly. We also see that the regulation of the sn2 position is more complex than at the sn1, and that PC exhibits stronger cooperative effects than PE. The distributions of fatty acids we analyze are the result of the homeostatic balance between the rates of biosynthesis, acyl-chain remodeling, and degradation, as well as the spectrum of fatty acids available. Since the samples we study are not connected by an explicit mechanistic process, our statistical analyses describe the aggregate effect of all processes, rather than distinguishing individual effects. A key aspect of our work is a novel statistically rigorous approach to determine whether chains at the sn1 and sn2 positions behave cooperatively based on contingency tables and Markov Chain Monte Carlo sampling. Thus by application of multiple computational approaches to lipidomic data we are able to determine the dependence between the sn1 and sn2 positions in choline and ethanolamine glycerophospholipids, quantifying the stringent molecular species regulation of these dominant lipid classes.

## Methods

### MDMS-SL quantification of lipids

Individual lipid extracts were reconstituted with 1∶1 (v/v) CHCl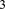/CH
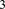
OH, flushed with nitrogen, and stored at 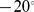
C prior to electrospray ionization-MS using a TSQ Quantum Ultra Plus triple-quadrupole mass spectrometer (Thermo Fisher Scientific, San Jose, CA) equipped with an automated nanospray apparatus (Advion Biosciences Ltd., Ithica, NY) and customized sequence subroutine operated under Xcalibur software. For each tissue type, 3–4 biological replicates were performed. C57BL/6J (B6) mice were euthanized at indicated ages during development or at 4 months of age. Tissues were excised, briefly washed in 10× diluted PBS and immediately freeze clamped using liquid nitrogen. All animal procedures were performed in accordance with the Guide for the Care and Use of Laboratory Animals and were approved by the Animals Studies Committee at Washington University School of Medicine.

### Identification of individual lipid molecular species

Lipidomic analysis was performed as previously described [4], [15], [19] and will be briefly described as follows. Both isobaric and isomeric species have the same nominal mass and therefore overlap in the spectrum acquired on a low to moderately high accuracy/resolution mass spectrometer. Isobaric lipid species are normally from different lipid classes and therefore have different head groups and thus different acyl composition (e.g., protonated 16∶1-22∶6 diacyl PE and 16∶0-18∶0 diacyl PC have different acyl composition but the same nominal mass of 762). Isomeric lipid species are from the same lipid class and therefore have identical acyl composition (e.g., 18∶0-18∶2 diacyl PE and 18∶1-18∶1 diacyl PE have identical acyl composition, i.e. total carbon number of 36 and total double bond number of 2).

In MDMS-SL, generally each lipid class or category is first selectively ionized through intrasource separation followed by a class-specific diagnostic scan to generate an ion peak list of the molecular species in a lipid class of interest for further acyl chain identification. This list is generated by matching the m/z values of the detected ion peaks in the diagnostic scan with those of the candidate species in the pre-established virtual database of the lipid class of interest. The list therefore contains information about the total number of carbon atoms and the total number of double bonds of the acyl chains. The presence of isobaric species, unlike the presence of isomeric species, does not affect acyl chain identification due to their differential total carbon number and total double bond number which discriminate them.

There are two types of isomeric lipid species, i.e., the acyl chain compositional isomers (e.g., 18∶0-18∶2 diacyl PE and 18∶1-18∶1 diacyl PE) and the regioisomers (e.g., 18∶0-18∶2 diacyl PE and 18∶2-18∶0 diacyl PE). The sum of the intensities of the paired acyl carboxylates in their corresponding PIS or in product ion scans in negative ion mode is used to assign the ratio between the acyl chain compositional isomers. For example, if the ratio of the sum of the intensities of 18∶0 and 18∶2 carboxylates to the intensity of 18∶1 carboxylate is 2∶1, then the 18∶0-18∶2 and 18∶2-18∶0 diacyl PE is 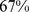, and the 18∶1-18∶1 diacyl PE is 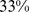.

The regioisomer ratio is assessed by the ratio of the intensities of the paired acyl carboxylates. The ratio of sn1 and sn2 acyl carboxylates in each lipid class or subclass is pre-determined by extensive examination of numerous product ion spectra of synthetic lipid standards with known sn1 and sn2 acyl chain composition. For example, the ratios of sn2 to sn1 acyl carboxylates are found to range from 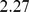 to 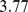 and center at 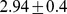 for molecular species in diacyl PE class (See File S1).

If the intensity ratio of 18∶2 carboxylate to 18∶0 carboxylate is 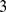 or larger in the above example, this PE species is identified as 18∶0-18∶2 diacyl PE and therefore the final assignment for this example is 18∶0-18∶2/18∶1-18∶1 at 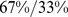. If the intensity ratio of 18∶2 carboxylate to 18∶0 carboxylate is 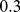 or less, this PE species is identified as 18∶2-18∶0 diacyl PE and therefore the final assignment is 18∶2-18∶0/18∶1-18∶1 at 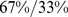. If the intensity ratio of 18∶2 carboxylate to 18∶0 carboxylate is between 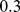 to 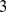, the regioisomers of 18∶0-18∶2 and 18∶2-18∶0 are both preseent. For example, if the intensity ratio is 2, the relative fractions of 18∶0-18∶2 and 18∶2-18∶0 are 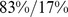 and the final assignment is 18∶0-18∶2/18∶2-18∶0/18∶1-18∶1 at 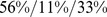. The details of the method as well as the effect of using a different threshold value (i.e., other than 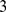) on the subsequent statistical analysis are discussed in File S1.

Two corrections are specifically considered for the determination of the intensities of carboxylates. One is the correction of the effect of 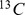 isotopologue on the carboxylate intensities as previously described [5]. Another is the correction for the reduced abundance of fatty acyl carboxylate containing multiple double bonds (e.g., 22∶6, 20∶4 and 18∶3 with a total double bond number of 3 or larger ) due to the facile loss of CO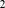 during collision induced dissociation (CID) in tandem mass spectrometry as previously demonstrated [4].

The analyzed spectroscopic molecular species datasets are provided in File S2. The relative abundances of isomers listed for a species were calculated as described above and annotated for mature heart, brain, lung, and liver. For the heart developmental data, the relative abundances of isomers were homogeneously estimated based on the described procedure. The relative abundances of isomeric species were approximated as 75%/25%, 64%/27%/9%, 50%/21%/21%/8% for di-, tri-, and tetra- isomers respectively.

### Positional independence model

Let 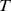 denote a phospholipid with two fatty acid chains (i.e. PC or PE). Assume that 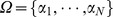 is the pool of possible fatty acid chain types that can be incorporated in either the sn1 or the sn2 positions of 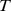. Incorporation of the fatty acid chains in the sn1 and the sn2 positions of 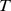 can be viewed as random processes, with random variables 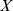 and 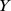 for the processes in the two respective positions. The joint random variable 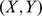 is distributed on the sample space 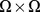.

Let 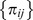 denote the joint probability 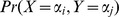, which can be obtained from the experimental concentration of the PC (or PE) species with chains 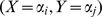 normalized so that they sum to 1. Denote the marginal probabilities by 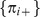 and 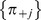, where 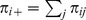 and 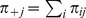. If the random variables 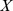 and 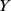 are independent, the joint probabilities should equal the product of their marginals. We wish to distinguish the null hypothesis of independence from the alternative hypothesis of cooperativity:
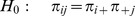


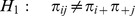
for all 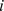 and 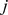. Sufficient statistics under the null hypothesis of independence are the column and row marginals.

To test the independence model, it is necessary to consider the measurement uncertainty in the 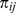 and determine whether deviations from 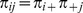 are statistically significant. For a given tissue type, the mean 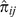 and standard deviation 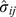 were calculated for each 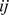 using the replicates for the tissue type. We modeled the variation in 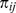 across replicates by a binomial process whose effective sample size would give the observed standard deviation. To determine this effective sample size, we calculated the variance effective sample size for each species 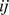 as
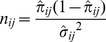
(1)We then averaged these values of 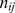 over all 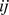 to get an effective sample size 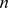 for the tissue type. For this averaging, outlier values of 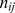 were excluded at the significance level of 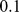, based on an assumed normal distribution for the 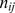 values. In the resulting model, probabilities 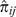 correspond to absolute counts 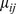 representing the total number of sampled molecules for each 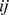 in the tissue type (see File S1).

For each tissue type, we then simulated 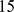 sets of values for the 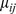 using 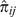 as weights and 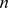 as the number of sampled molecules with a multinomial sampling process. For each of these simulated sets, the positional independence model was then evaluated by performing the Fisher's exact test. The p-value in the Fisher's test was calculated by summing the probabilities of all permissible tables with equal or more extreme arrangement than the observed table [20], [21]. In cases where 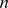 is large, it is impractical to enumerate all possible tables. Thus Markov Chain Monte Carlo (MCMC) was used to explore the space of permissible tables and approximate the p-value [21]. In our implementation, we used the R package aylmer.test and 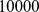 permissible tables were generated in the MCMC simulations to calculate the p-values. In the MCMC procedure, cells with zero counts were kept fixed. For each tissue type, the p-value for independence was reported as the average of the p-values of the 15 simulated sets.

For testing of the quasi-independence model, we selected subsets of chain types at the sn1 and sn2 positions. The subsequent statistical test was performed on the partial frequency table as described above.

### Clustering Analysis

The conditional probability 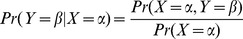 describes the probability of observing chain type 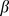 at the sn2 position given that chain type 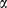 is at the sn1 position. We performed clustering on the conditional distributions to determine whether there are acyl chains in the sn1 position that influence chains at the sn2 position in the same way. To do this clustering, for every pair of sn1 chain types we measured the distance between their sn2 conditional distributions, 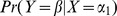 and 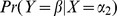, using Jensen-Shannon Divergence. We then performed hierarchical clustering on the distance matrix. By examining the clustering from all tissue types, which showed similarities, we defined a set of canonical/noncanonical chains types at each of the sn1 and sn2 positions.

In the test of quasi-independence, we selected a set 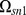 of the 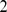 most similar sn1 canonical chain types for a given tissue type. A second hierarchical clustering of sn2 canonical chain types was performed based on sn1 conditional distributions, i.e., 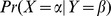 for 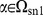. All pairs of clustered sn2 chain types 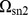 were considered. Tests of independence were performed on these subsets. For any subset where the null hypothesis was not rejected at threshold p-value 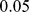, we continued searching for a larger subset by including the next closest sn1 or sn2 chain types.

### Deviation from Independence

The deviation of each 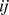 from the independence model was quantified by the standardized residual
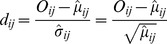
(2)where 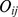 denotes the observed frequency and 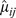 denotes the expected frequency of the cell 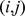. Note that here, the counts in the table are modeled as realizations of an independent Poisson random variable. This is equivalent to testing the hypothesis of independence in a multinomial model [20]. See File S1 for details.

### Jensen-Shannon Divergence

Jensen-Shannon Divergence (JSD) is a statistical measure of the divergence of two or more probability distributions. We used JSD to compare multiple phospholipid distributions as:
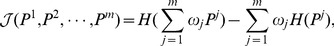
(3)


Here 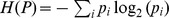 is the Shannon entropy, 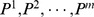 are the probability distributions, and 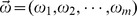 is a vector of weights with 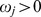, 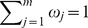. For the comparison of the observed and the independence distributions, 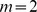 and the weights are assigned uniformly i.e., 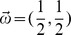. For comparing the observed distribution across the tissues (mature heart, brain, lung, and liver), 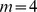 and the weights are assigned equally as well, i.e., 
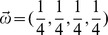
.

For the comparisons of the marginal distributions across time points, the weights were scaled to be proportional to the interval between the successive time points, i.e. 
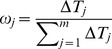
. More specifically, given time points 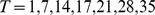, the computed weights were 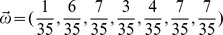. This scaling allows us to take the timespans at which the lipid concentrations were measured into consideration and attain an unbiased evaluation of the changes in the molecular species profiles.

## Results

In this work, we statistically analyzed lipidomic data for PC and PE in 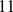 types of mouse tissues. These were brain, heart, lung, and liver from 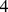 month old C57BL/6J(B6) WT mice; and developing heart from C57BL/6J(B6) WT mice at 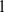, 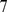, 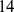, 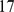, 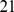, 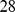, and 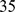 days of age [15]. Each tissue type was measured in 3–4 biological replicates. For each tissue type, the probability distribution for lipid molecular species was calculated by normalizing concentrations to sum to one and averaging among replicates. This yielded probabilities for molecular species with X at the sn1 position and Y at the sn2 position 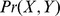.

### Characterization of processes controlling species composition from sn1 and sn2 marginal distributions

The distributions of fatty acids at the sn1 and the sn2 positions of phospholipid provide valuable information about the processes controlling species composition. The marginal distribution at the sn1 position quantifies the acyl composition at the sn1 irrespective of the sn2, and vice versa for the marginal distribution at the sn2 position. For each tissue type and lipid class we calculated the marginal probability of observing chain 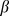 at the sn2 position as 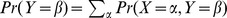 as well as the marginal probability of observing 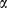 at the sn1 position 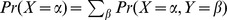.

Comparing PC and PE marginal distributions, we found that they are generally similar. For both types of molecules, the predominant fatty acids at the sn1 position are 16∶0 and 18∶0, while the fatty acids at the sn2 position are generally the longer and unsaturated fatty acid chains 18∶2, 20∶4, and 22∶6. This is consistent with many previous findings [22]–[24]. However, PC and PE marginal distributions display different levels of similarities across samples.

**Figure 1:**
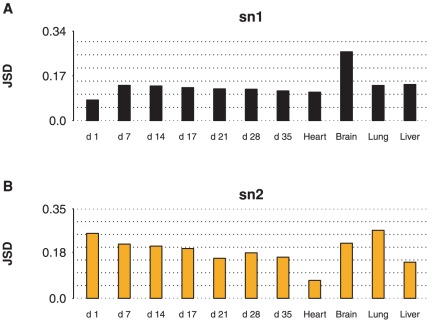
Jensen-Shannon divergence of PC and PE marginal acyl distributions. For each of sn1 (A) and sn2 (B) positions, the dissimilarity between PC and PE marginal acyl distributions was measured by Jensen-Shannon Divergence (JSD). The highest similarity was observed for mature heart sn2 (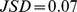), while the lowest similarity was observed for mature lung sn2 (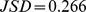). A trend of decreasing JSD was observed during heart development from day 7 to day 35, for both sn1 (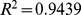, 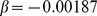) and sn2 (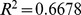, 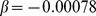).

**Figure 2:**
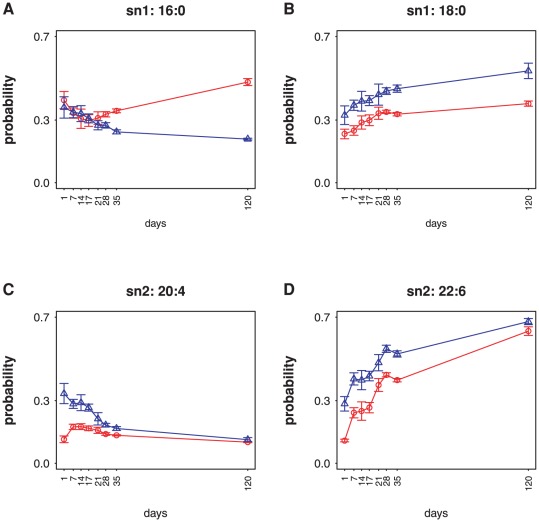
PC and PE acyl chain marginal probabilities during heart development. Trajectories of marginal probabilities of PC (red circles) and PE (blue triangles) for sn1 16∶0 (A), sn1 18∶0 (B), sn2 20∶4 (C), and sn2 22∶6 (D). PC and PE marginal probabilities closely follow each other in B, C, and D, suggesting these chain types are substrates of these two phospholipids' shared processes controlling species composition. The two curves in A are divergent, indicating that acyl chain regulation of sn1 16∶0 in PC and PE differs.

To quantify similarities between PC and PE, we measured Jensen-Shannon Divergence (JSD) between PC and PE marginal distributions for each sample and at each sn position. JSD measures the dissimilarity between probability distributions symmetrically with values ranging from 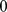 (when distributions are identical) to 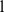 [25]. PC vs. PE comparisons for all samples are shown in Figure 1. In general, PC and PE are more similar at the sn1 position than at the sn2 position. In 9/11 tissues, the sn1 JSD is lower than the sn2 JSD. The exceptions are for mature heart and mature brain. The sn2 acyl distributions of mature heart PC and PE have the lowest JSD of any comparison (JSD = 0.070). This is likely due to the presence of the dominant 20∶4 and 22∶6 acyl chains which tend to highly localize in the sn2 position in both PC and PE. However, for mature brain, the sn1 acyl distributions have the second highest JSD of any comparison (JSD = 0.261), likely because of the presence of more monounsaturated acyl chains (e.g. 18∶1) in brain PC than in brain PE. It is worth noting that for all comparisons, the JSDs are low on an absolute scale (max. 0.266 for sn2 lung). This indicates that, despite some small variations by tissue type, PC and PE are largely regulated by similar processes (also see Figure 2). Thus, PC and PE appear to be governed by a common set of constitutive acyl chain regulatory processes, with distinctions from this behavior occurring only occasionally in specific tissue types.

Interestingly, we observed a trend of PC and PE becoming more similar during heart development from day 7 to day 35. This can be seen by the decrease in JSD with time at both the sn1 (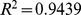) and sn2 (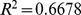) positions. However, the mechanisms leading to this increased similarity of PC and PE differ by position. The slope of change at the sn2 position (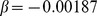) is much smaller than that at the sn1 position (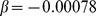). It is notable that the day 1 sn1 JSD is unusually low relative to the other sn1 timepoints. This is likely due to stronger maternal bias and the presence of dominant nascent immature species containing less unsaturated acyl chains at day 1, with subsequent days reflecting the processes controlling species composition activated during development.

To better understand the processes that distinguish PC and PE, we examined the trajectory of marginals during heart development. Figure 2 shows the PC and PE marginals for two abundant chain types at the sn1 position (16∶0, 18∶0) as well as the two abundant chain types at the sn2 position (20∶4, 22∶6). The general similarity in processes controlling PC and PE species composition can be seen in the trajectories for the sn1 18∶0, sn2 20∶4, and sn2 22∶6 marginals. All 3 of these marginals track closely between PC and PE, suggesting that they are substrates of the two phospholipids' shared regulatory processes. However, the PC and PE sn1 16∶0 curves diverge during development. This suggests that a process specific to 16∶0 chains in the sn1 position becomes active during heart development, and that this process acts distinctly for PC and PE. Thus comparative consideration of marginals can be used to reveal the existence of tissue- and acyl-specific regulatory processes.

### Characterization of processes controlling species composition from sn1-sn2 conditional distributions

**Figure 3:**
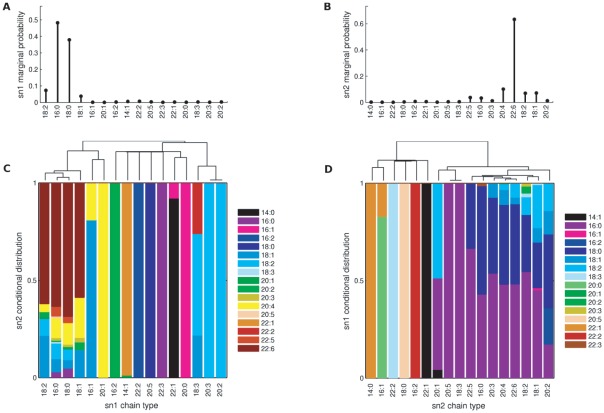
Conditional distributions and clustering of acyl chains. For PC mature heart, a set of sn1 chain types 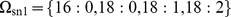 (C) and a set of sn2 chain types 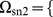
16∶0, 18∶1, 18∶2, 20∶3, 20∶4, 22∶5, 22∶6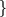 (D) were each clustered. Within each of these clusters, conditional acyl distributions were highly homogeneous. It is interesting to note that at each of the sn1 and sn2 positions, the most abundant chains (panels A and B) tend to be clustered together (panels C and D), indicating similar conditional effects.

We next investigated cooperative effects between sn1 and sn2 positions, i.e. whether a particular chain at one position influences the distribution of chains at the other position. To evaluate association between the positions, we considered the conditional probability 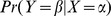, i.e. the distribution of chains 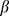 at the sn2 position given chain 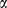 at the sn1 position. Figure 3(C) shows conditional distributions of the sn2 chains given the sn1 chain for PC mature heart. These conditional distributions vary greatly depending on the sn1 chain. For example, when the sn1 chain is 18∶2, the major sn2 chain is 22∶6. However, when the sn1 chain is 18∶3, the predominant sn2 chain is 18∶2. This suggests that for specific class of sn1 chains, the enzymes involved in regulatory processes at the sn2 position also interact with the acyl chain at the sn1 position.

To investigate the similarity among these conditional relationships, we clustered the sn2 distributions 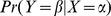 as a function of the sn1 chain (see Methods). Interestingly, a group of sn1 chain types, 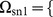
16∶0, 18∶0, 18∶1, 18∶2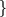 display very similar conditional sn2 distributions. For these sn1 chains, the predominant sn2 chain is always 22∶6, with smaller but consistent contributions from 20∶4 and 18∶1. Thus, while enzymes that regulate the acyl chain at the sn2 position also recognize the acyl chain at the sn1 position, they are indifferent among the sn1 chains in the set above.

We also considered the dependence 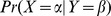, with results for PC mature heart shown in Figure 3(D). As expected, we observed that the sn2 chain affects the distribution at the sn1 position (contrast Y = 16∶1 versus Y = 22∶6). However, we also saw groups of sn2 chains with comparable influence on the sn1 position. Clustering of 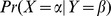 by 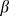 results in a set of chains 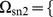
16∶0, 18∶1, 18∶2, 20∶3, 20∶4, 22∶5, 22∶6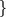 with similar sn1 conditional distributions.

Similar behaviors were observed for all the tissue types studied, although the particular conditional patterns vary from one sample to another (see File S1 for clustering figures). Based on these observations, we defined universal canonical chain types, 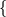
16∶0, 16∶1, 18∶0, 18∶1, 18∶2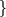 for sn1 and 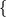
16∶0, 18∶1, 18∶2, 20∶0, 20∶1, 20∶3, 20∶4, 20∶5, 22∶3, 22∶4, 22∶5, 22∶6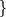 for sn2 respectively, which tend to cluster together across samples. Remarkably, for most samples, the canonical chain types include the most abundant chain types at the given position, such as 16∶0, 18∶0 for sn1 and 18∶2, 20∶4, 22∶6 for sn2. 16∶0 and 18∶0 are among the three most abundant chain types at the sn1 position for every tissue type and lipid class. 20∶4 and 22∶6 are among the three most abundant chain types at the sn2 position for 9/11 tissue types for PC and 11/11 tissue types for PE. In general, there is only weak association between the sn1 and the sn2 acyl chains when the most abundant canonical chain types are considered, but for noncanonical chain types we observe stronger associations.

**Figure 4:**
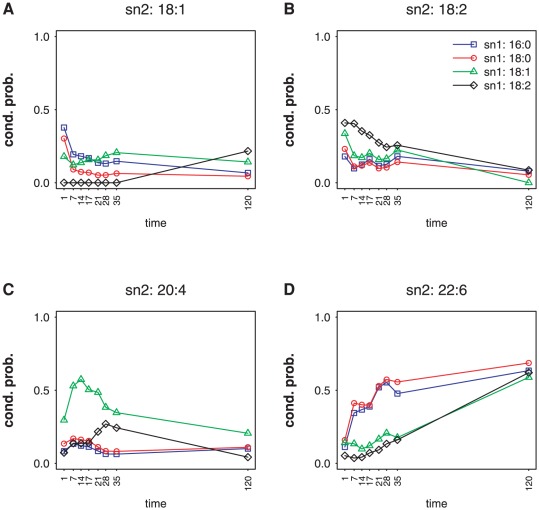
PC acyl chain conditional probabilities during heart development. The sn2 conditional probabilities given sn1 chain types, 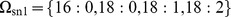 closely follow each other. Shown are conditional probabilities for the four major sn2 chain types 18∶1 (A), 18∶2 (B), 20∶4 (C), and 22∶6 (D).

Figure 4 shows the trajectories of the conditional probabilities 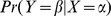 of the four major sn2 chain types: 18∶1, 18∶2, 20∶4, 22∶6, given sn1 (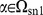) for PC during heart development. Although these conditional probabilities show distinct behaviors as a function of 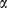 at day 1, they converge by the time the mouse reaches maturity (day 120). In particular, the distributions 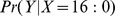 and 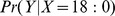 show a striking similarity, as these values (red and blue dots) are very close at all developmental timepoints and for all sn2 chains. Similar behavior was observed for PE trajectories (see File S1). 16∶0 and 18∶0 are notable because of their common biochemical characteristics of similar length and lack of unsaturated bonds. These data indicate that the enzymes involved in controlling species composition are indifferent among adjacent acyl chains with similar biochemical properties. We will examine the extent of this indifference in the next section.

### Quantifying independence between sn1 and sn2 chains

**Figure 5:**
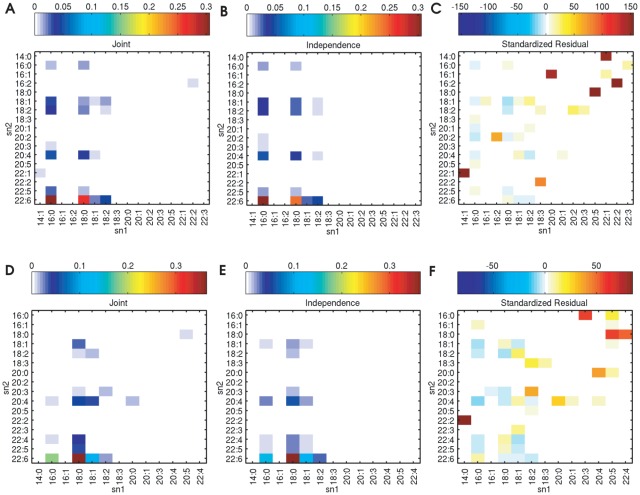
Comparison of joint and product distributions for mature heart. Heatmaps of the joint distribution (*l*eft), the independent product distribution (*m*iddle), and their standardized residual (*r*ight) for mature heart PC (*t*op) and PE (*b*ottom). The joint and the product distributions are similar, suggesting that the acyl chains at the sn1 and the sn2 positions are largely independent.

While the previous section suggests the existence of sn1-sn2 dependencies for acyl chains with different biochemical properties, the hypothesis that sn1 and sn2 acyl chains influence one another has not been directly tested. We can do this by comparing the experimentally observed joint 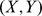 probability distribution for PC (or PE) to the product of the sn1 and sn2 marginal distributions. Figure 5 shows heatmaps of the joint and product distributions as well as their standardized residuals for the mature heart PC and PE. The qualitative similarity between the joint and the product distributions suggests that sn1 and sn2 chains will be generally independent.

To formally test independence, we designed a novel, statistically rigorous approach based on the Fisher's exact test. For the Fisher's test it is necessary to use counts rather than probabilities. Based on the mass of lipids analyzed, the number of molecules of PC or PE in each sample can be estimated, which could in principle be used to scale probabilities to counts. However, counts on this scale would be computationally infeasible for a Fisher's test, and this problem would be exacerbated by the disparate concentrations of species. Therefore we instead mapped probabilities to counts using the variance effective sample size (see Methods). This approach effectively makes use of the experimentally observed variation in the data while allowing a reasonable computation time.

Note that our Fisher's-based approach is necessary because more commonly used tests of independence such as the Pearson's 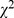 test are unreliable on this type of data. Table 1 shows an example of the contingency table of mapped counts for PC mature heart sample using the canonical chains. As can be seen, the table contains many species with zero counts. The Pearson's 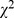 test of independence will not produce reliable p-values for data with such zero counts. This is because the accuracy of the 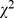 test is dependent on asymptotic behavior of the Pearson's 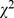 test statistics, but this behavior often fails when counts are low. The Fisher's exact test provides an accurate alternative not affected by such small counts. Another important consideration is that the variance effective sample size 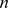 is in general still large, making it impractical to enumerate the entire permissible tables. Therefore we used Markov Chain Monte Carlo (MCMC) to explore the space of permissible tables and approximate the p-value.

**Table 1:** PC mature heart.

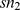												
		16∶0	18∶1	18∶2	20∶0	20∶1	20∶3	20∶4	20∶5	22∶4	22∶5	22∶6
	16∶0	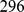	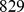	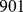	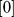	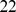	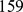	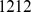	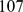	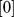	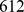	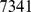
	16∶1	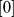	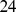	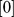	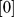	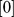	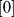	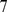	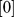	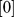	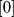	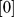
	18∶0	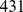	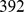	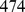	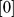	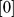	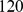	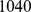	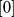	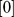	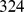	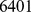
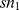	18∶1	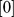	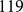	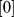	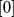	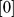	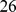	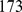	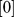	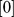	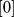	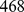
	18∶2	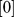	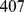	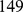	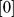	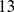	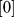	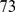	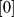	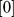	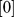	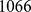

Two-way Contingency table for mature heart PC sample. The structural zeros are denoted by 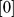. The variance effective sample size is 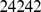.

Our results indicate the presence of cooperative effects between the sn1 and sn2 positions in all tissue types when all chains are considered. For each tissue type, we performed the Fisher's exact test to test the null hypothesis of independence of sn1 and sn2 positions, with zero count cells kept fixed. We found that the null hypothesis of independence was rejected at cut-off p-value of 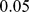 for all tissue types when including all chain types in the analysis. This indicates the presence of cooperative associations.

Given the general independence of sn1 and sn2 positions suggested by Figure 5, we hypothesized that in each tissue the sn1 and sn2 position would be “quasi-independent”, i.e. there would be a few dependencies between individual classes of acyl chains, but there would also be groups of chains for which the sn1 and sn2 positions are independent. To determine groups of chains with such independence, we considered the sn1 and sn2 chains in the canonical clusters 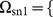
16∶0, 16∶1, 18∶0, 18∶1, 18∶2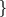 and 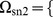
16∶0, 18∶1, 18∶2, 20∶0, 20∶1, 20∶3, 20∶4, 20∶5, 22∶3, 22∶4, 22∶5, 22∶6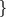. For each tissue type, we then tested whether there are sufficient statistical evidence for independence among subsets of chains in these canonical clusters.

To find these subsets we first selected the 2 closest sn1 canonical chain types, based on their clusterings described in the previous section. We then performed the test of independence on these 2 sn1 chains against all possible pairs of clustered canonical sn2 chain types. For any subset where the null hypothesis was not rejected (significance level 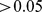), we continued searching for a larger subset by adding the next closest sn1 or sn2 chain types and then testing independence again. In all tissue types, we found at least one subset of sn1 and sn2 chain types for which the independence hypothesis was not rejected. The subsets contributing the most to the total concentrations are reported in Tables 2 and 3.

**Table 2:** Test of quasi-independence on PC molecular species distributions.

Sample Name	Sample Size	sn1 FA	sn2 FA	p-value	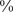 of Total Species
heart day 1	5949	16∶0, 18∶0	18∶2, 20∶3, 20∶4, 22∶5, 22∶6	0.0637	31.91 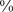
heart day 7	11148	16∶0, 18∶0	18∶0, 18∶2, 20∶4, 22∶6	0.1090	38.10 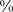
heart day 14	6220	16∶0, 18∶0	18∶0, 18∶2, 20∶4, 22∶6	0.0724	39.80 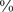
heart day 17	9333	16∶0, 18∶0	18∶2, 22∶6	0.3228	32.32 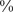
heart day 21	3900	16∶0, 18∶0	18∶2, 20∶4, 22∶6	0.1013	46.89 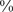
heart day 28	12170	16∶0, 18∶0	20∶4, 22∶6	0.0835	42.57 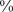
heart day 35	49212	16∶0, 18∶0	20∶4, 22∶6	0.0799	39.57 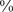
heart 4 mon	24242	16∶0, 18∶0	20∶4, 22∶6	0.4948	65.58 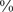
liver 4 mon	5955	16∶0, 18∶0, 18∶1	20∶3, 20∶4, 20∶5, 22∶6	0.1832	42.92 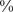
lung 4 mon	32310	16∶0, 18∶0	18∶1, 18∶2	0.2506	28.85 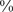
brain 4 mon	4567	16∶0, 18∶0	18∶1, 18∶2, 20∶3, 22∶4	0.7338	28.64 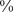

For each sample, we identified subsets of sn1 and sn2 fatty acids (FA) for which the subsets passed the independence test (p
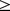
0.05). The 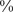 of Total Species indicates the total fraction of PC made up by species in the independent set for the tissue type.

**Table 3:** Test of quasi-independence on PE molecular species distributions.

Sample Name	Sample Size	sn1 FA	sn2 FA	p-value	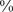 of Total Species
heart day 1	2178	18∶0, 18∶1	18∶1, 18∶2, 20∶4, 22∶3, 22∶4, 22∶6	0.1860	43.37 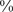
heart day 7	6461	18∶0, 18∶1	18∶1, 20∶0, 20∶4, 22∶4, 22∶6	0.3197	43.23 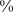
heart day 14	4640	18∶0, 18∶1	18∶1, 18∶2, 20∶0, 20∶4, 22∶3	0.4231	24.91 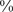
heart day 17	6452	18∶0, 18∶1	18∶2, 20∶0, 20∶4, 22∶4, 22∶6	0.0652	46.18 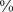
heart day 21	3471	18∶0, 18∶1	18∶1, 18∶2, 20∶0, 20∶1, 20∶4, 22∶4, 22∶6	0.3723	50.73 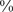
heart day 28	7010	18∶0, 18∶1	18∶1, 22∶3, 22∶5	0.7417	12.74 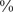
heart day 35	9878	18∶0, 18∶1	18∶2, 20∶0, 20∶1, 20∶4, 22∶4, 22∶6	0.0517	52.39 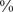
heart 4 mon	7204	16∶0, 18∶0	20∶4, 22∶4, 22∶5	0.1326	14.27 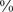
liver 4 mon	28197	16∶0, 16∶1	18∶2, 20∶4	0.3516	12.08 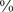
lung 4 mon	26989	16∶0, 18∶1	18∶1, 18∶2, 20∶4, 22∶4, 22∶6	0.0545	45.34 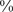
brain 4 mon	26242	18∶0, 18∶1	20∶4, 22∶5	0.4927	26.14 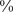

For each sample, we identified subsets of sn1 and sn2 fatty acids (FA) for which the subsets passed the independence test (p
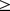
0.05). The 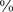 of Total Species indicates the total fraction of PE made up by species in the independent set for the tissue type.

For example, in mature heart data, the sn1 fatty acids 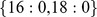 and sn2 fatty acids 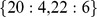 meet the independence test, and these species contribute 65.58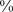 of the total PC concentration. Therefore processes controlling species composition in this tissue appear to be a mixture of position-independent and cooperative mechanisms with independence being the more common behavior. This analysis confirms the findings in the previous section that chains with similar biochemical properties (length and saturation) have a similar effect on the acyl chain remodeling of other chains in the molecule. For PC, the shorter saturated chains 16∶0 and 18∶0 often group at the sn1 position, and the longer unsaturated chains 20∶4 and 22∶6 frequently group at the sn2 position.

### Trends in processes controlling species composition

**Figure 6:**
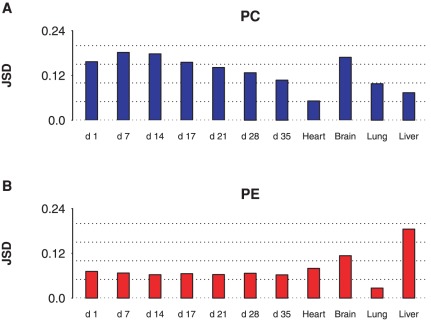
Deviation from independence, as a function of tissue type. The overall deviation of the joint distribution from the expected independent distribution was scored using JSD for PC (A) and PE (B). The lowest JSD was observed in lung PE (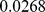), while the highest was observed in liver PE (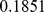). During heart development from day 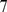 to day 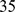, a trend of decreasing JSD was observed in PC (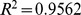, 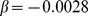), while PE displayed stable JSD during the time period, (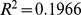, 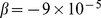).

The divergence of the observed species distribution from the predicted independent distribution provides a useful quantification of how independent the sn1 and sn2 positions are. We used Jensen-Shannon Divergence to quantify the difference between two distributions (see Methods). Figure 6 shows the JSD of the observed joint distribution when compared to the independent distribution for PC and PE and for each sample. Lung PE has the lowest JSD (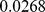), indicating strong independence of the processes controlling species composition at the sn1 and sn2 positions. Liver PE has the highest JSD (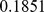), indicating greater cooperativity.

We also observed systematic trends in species composition. During heart development, PC JSD decreases from days 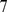 to 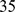 (linear fit: 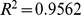, 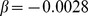). This indicates that processes that act on the sn1 and sn2 positions independently become increasingly important over time. However, there is no systematic change in PE JSD during development (linear fit: 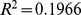, 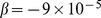). During heart development, PE JSD is lower than PC JSD at every time point, though mature heart PE JSD is slightly larger than that of PC JSD. PE is also more independent in mature brain and lung, though PE appears to be subject to unusually extensive sn1 and sn2 cooperativity in liver.

### Comparing complexity of sn1 and sn2 acyl chain remodeling

Which sn position is controlled by more complex regulation? We hypothesized that if the regulatory mechanisms on one position are more complex, the acyl chain distribution at that position should display greater variation across conditions. This is analogous to how the expression of highly regulated genes varies widely across conditions while the expression of housekeeping genes is constitutive. For instance, if there is greater temporal regulation of acyltransferase/transacylase activity at one position, one would expect to see more variability in the distribution of acyl chains at that position through time. Similarly, if anatomy-specific regulation is greater at one of the positions, the distribution of the acyl chains at that position should show more variability across tissues. On the other hand, if the regulation scheme is simpler at one position, then we expect to see a more stable acyl chain distribution across conditions. We calculated and compared the variation in sn1 and sn2 marginal distributions across conditions using a multi-distribution Jensen-Shannon Divergence (higher JSD corresponds to more variation).

**Figure 7:**
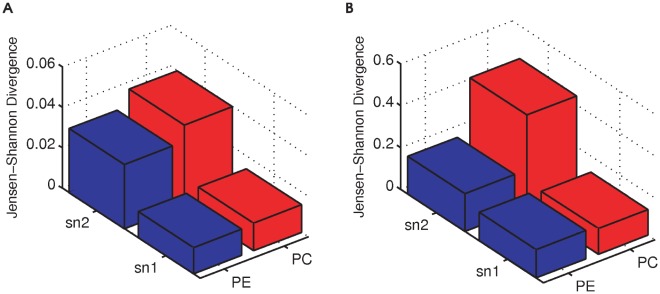
Comparing complexity of sn1 and sn2 acyl chain remodeling. For each of PC sn1, PE sn1, PC sn2, PE sn2, the variation of their marginal distributions across conditions was quantified using a multi-distribution JSD. The sample conditions in (A) are heart development time points: day 1, 7, 14, 17, 21, 28, 35. The sample conditions in (B) are four different anatomies: mature heart, brain, lung, and liver. We found that the sn2 position varies more than sn1, suggesting sn2 positions are more subject to regulation than sn1. This conclusion is consistent for each of PC and PE for both temporal variation and anatomical variation.

Figure 7 shows the JSD for marginal distributions during heart development. The sn1 position exhibits less variation than the sn2 in PE (blue). This is also the case for PC. Similarly, the JSD of the marginal probabilities across different anatomies (mature heart, brain, lung, and liver) indicates a higher variability of the sn2 position than in the sn1 position for both PC and PE. According to the above hypothesis, these data indicate that sn2 positions are subject to more complex regulation than the sn1. Interestingly, the PE JSD values at the sn2 position are noticeably lower than the PC JSD values at the sn2 position (PC sn2 has the highest JSD). This is consistent with the prior observation that PC exhibits stronger sn1-sn2 cooperativity than PE. Stronger cooperative effects allow for finely tuned regulation of PC species distributions. However, the weaker sn1-sn2 cooperativity in PE is likely to be less functionally important, since PE species distributions do not appear to be as regulated.

## Discussion

Using novel statistical approaches, we have identified the major dependence relationships between sn1 and sn2 acyl chains in PC and PE, the two most common phospholipids in eukaryotic membranes. While the mechanisms which determine acyl distributions (synthesis, degradation, and acyl chain remodeling) generally do not yield sn1-sn2 cooperativity, their effects are clearly position-specific. This behavior is different from the tetra-acyl phospholipid cardiolipin (CL) [17]. For CL, it was shown that the tetra-acyl distributions in most tissues were well-fit by a model in which the four positional acyl distributions were identical and lacked cooperativity, and that a model that allowed distinct behavior between sn1 and sn2 positions did not fit the data better. PC and PE are notably different from CL for their strong distinction in sn1 and sn2 behavior.

Cooperative effects between sn1 and sn2 acyl chains are more apparent for low abundance species, while canonical chains with high abundance generally exhibit independence between sn1 and sn2 positions. The observed cooperative effects are not due to variation among the biological replicates, which are taken into account by the Fisher's test. However, it should be noted that in the case of minor species, the identification of regioisomers is sensitive to uncertainties in sn1 and and sn2 relative carboxylate formation during the mass spectrometry procedure, which in turn may affect the test of independence (see File S1 for full details).

On the other hand some of the low abundance species consistently deviate from independence model across tissues and they tend to be more abundant than expected from the independence model. The consistency in the deviation is statistically significant, suggesting that the observed cooperativity might arise from preferential recognition by regulatory enzymes (see File S1 for full details). If correct, we speculate that the low abundance, cooperatively regulated phospholipid species may provide specialized functions for particular tissues or at a particular developmental stages, analogous to the manner in which highly regulated gene expression leads to precise tissue- or timing-specific enzyme production. Such phospholipid compositional regulation may also influence properties such as signaling scaffolds, membrane fluidity, and energy metabolism in a tissue- and timing- specific manner. This provides a mechanism for how lipid regulatory enzymes controlling species composition may play key roles in individualized biological functions.

The enzymes responsible for these cooperative effects remain an important area for further study. In mammalian cells, it is known that PC is synthesized predominantly via the CDP-choline pathway, and the PE N-methyltransferase (PEMT) pathway accounts for 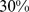 of synthesis in liver and a smaller fraction in other tissues [26], [27]. In liver, PC species with mono- or di-unsaturated sn2 acyl groups such as 16∶0-18∶1/16∶0-18∶2 are preferentially created in the CDP-choline pathway [26]. Similarly in heart, PC species with mono-unsaturated acyl groups at their sn2 position are preferentially synthesized, regardless of the chain type at the sn1 position (e.g., 18∶0-18∶1/18∶1-18∶1/16∶0-18∶1) [28]. Meanwhile, the PEMT pathway shows no substrate specificity for PE, phosphatidyl-N-monomethylethanolamine, or phosphatidyl-N,N-dimethylethanolamine [29], although modest specificity for 16∶0-22∶6/18∶0-22∶6 has been reported [26]. In our data, we did not observe a preferential enrichment of 16∶0-22∶6/18∶0-22∶6 in liver PC or a depletion in liver PE. This suggests that in our liver samples the PC acyl chain remodeling processes have overwritten the distributions created during PEMT synthesis. The biosynthesis of PE involves the CDP-ethanolamine (Kennedy) pathway and the decarboxylation of phosphatidylserine (PS) [30], [31]. These two alternative routes contribute differently to overall PE synthesis in different tissues. For example, in liver and heart, the CDP-ethanolamine pathway was reported to produce the majority of PE, whereas in many other types of cells PS decarboxylase makes 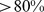 of PE [32], [33]. It has also been shown that these two pathways generate different PE molecular species [34]. The CDP-ethanolamine pathway preferentially synthesizes PE species with mono- or di-unsaturated fatty acids at sn2 position, (e.g., 16∶0-18∶2/18∶1-18∶2). The PS decarboxylation pathway preferentially generates PE species with polyunsaturated fatty acids at sn2 position such as 18∶0-20∶4 [34]. We did observe slight enrichment of both PE 18∶1-18∶2 and 18∶0-20∶4 in liver, implying modest dependencies between the fatty acids in sn1 and sn2 positions in PE synthesis.

After de novo synthesis, PC and PE acyl chain remodeling involves transacylation or deacylation by PLA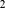 or PLA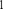, followed by reacylation mediated by various acyltransferases. Although acyl chain remodeling behaviors have been investigated primarily for only one sn position at a time, cooperative effects have not been extensively observed [22], [35], [36]. This fact supports a quasi-independence model, i.e. sn1 and sn2 positions are independent for canonical high abundance species, with cooperative effects important primarily for low abundance species. A more subtle possibility is that there may be cooperative effects for high abundance species, but that all high abundance species are subject to the same effects. A study by Kazachkov et al supports this idea, as they observed that the activity of the sn2 acyl transferase LPCAT3 was influenced by the chain at the sn1 position, but all saturated sn1 chains (which are more abundant) yielded higher activity than unsaturated sn1 chains [37]. What controls the abundance of noncanonical species in membranes is an important question for further study. In addition to acyl transfer and transacylation, direct chemical modifications and transport may also play a role [38].

Our method can set the stage for elucidating the processes controlling lipid species composition using large-scale lipidomic measurements. Of particular interest are pulse-chase timecourse experiments that provide measurements of metabolism of heavy-isotope labeled glycerophospholipid species [9], [39]. These approaches provide highly detailed information on remodeling of exogenously added glycerophospholipids. While prior studies have shown qualitatively that remodeling rates at the sn1 and sn2 positions depend on the saturation states of the chains at the two positions [39], accurate determination of the network of acyl chain flux and inference of the acyl chain remodeling processes from these comprehensive and complex datasets are challenging tasks. The results we have presented here indicate that sn1/sn2 independence can be assumed for most high abundance chains, with deviations only for minor species. This finding suggests that the complexity of the dynamical system that governs phospholipid remodeling can be greatly simplified from one in which there are frequent sn1-sn2 dependencies. This will significantly reduce the number of parameters to be inferred. Since the rate parameters of these reactions are unknown a priori, reduction of the complexity is an important step in computational inference of the remodeling processes (manuscript in preparation).

Our method also suggests it will be feasible to analyze the essential acyl chain remodeling behaviors in other acyl-containing phospholipids, such as phosphatidylglycerol (PG), phosphatidylserine (PS), phosphatidylinositol (PI), phosphatidic acid (PA), and acyl-containing glycerolipids such as triacylglycerol (TAG), as well. Integrating these species together into a lipid synthesis and acyl chain remodeling network will be an important challenge. There is a growing requirement to develop further bioinformatics tools for the analysis of high throughput mass spectrometry lipidomic data [40]. Statistical models for such data will be vital for elucidating disease mechanisms that act via the lipidome.

## Supporting Information

File S1 Text and figures for a additional analysis and tests.
(PDF)

File S2 Data file of the measured concentrations of lipid molecular species.
(TXT)
