# Supplementary material for: Statistical Analysis of the Processes Controlling Choline and Ethanolamine Glycerophospholipid Molecular Species Composition
Source: PLoS One. 2012 May 25;7(5):e37293. doi: 10.1371/journal.pone.0037293 (PMC3360702; doi:10.1371/journal.pone.0037293)
Supplement: File S1 — Text and figures for a additional analysis and tests. (PDF) [file pone.0037293.s001.pdf]

Supplementary Information
Statistical analysis of the processes controlling choline and ethanolamine glycerophospholipids molecular species composition

Kourosh Zarringhalam[1,†], Lu Zhang[1,†], Michael A. Kiebish[2], Kui Yang[2], Xianlin Han[3], Richard W. Gross[2], and Jeffrey Chuang[1,*]

1 Department of Biology, Boston College, Chestnut Hill, MA

2 Department of Internal Medicine, Division of Bioorganic Chemistry and Molecular Pharmacology, Washington University School of Medicine, St. Louis, MO

3 Sanford Burnham Medical Research Institute, Diabetes and Obesity Research Center, Orlando, FL

† These authors contributed equally.

∗ E-mail: chuangj@bc.edu

Contents

1 Evaluation of the uncertainties in identification of regioisomers 2

2 Variance effective sample size 7

3 Determination of species causing deviations from the independence model 10

List of Tables

1 Concentration and the ratios of isobaric species. 4
2 Test of quasi-independence on PE molecular species for alternative regioisomer assignments. . 6
3 Scheme for the analysis of deviated species across tissues. 10
4 Deviated species. 11

List of Figures

1 PE sn2/sn1 ratios. 2
2 Concentration of the $[\alpha, \beta]$ isomer. 3
3 Clustering of PE for alternative regioisomer assignments. 5
4 Conditional probabilities of PE during heart development. 8
5 Clustering of PC sn1 chain types. 9

1 Evaluation of the uncertainties in identification of regioisomers

Regioisomers are isomeric lipid species with identical chain types but at different sn positions (e.g., 18:0-18:2 diacyl PE and 18:2-18:0 diacyl PE). The regioisomer ratio is assessed by the ratio of the intensities of the paired acyl carboxylates. The ratio of sn1 and sn2 acyl carboxylates in each lipid class or subclass is pre-determined by extensive examination of numerous product ion spectra of synthetic lipid standards with known sn1 and sn2 acyl chain composition. The ratios of sn2 to sn1 acyl carboxylates range from 2.27 to 3.77 and center at 2.94 ± 0.4 for molecular species in diacyl PE class (See Figure 1).

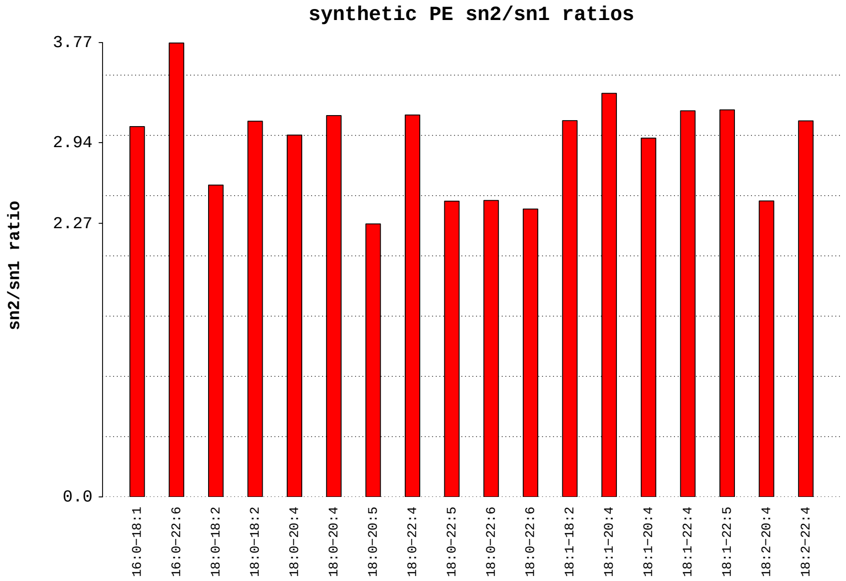

Figure 1: PE sn2/sn1 ratios. The ratio of sn1 and sn2 acyl carboxylates in each lipid class or subclass is pre-determined by extensive examination of numerous product ion spectra of synthetic lipid standards with known sn1 and sn2 acyl chain composition.

The regioisomer assignment was performed as follows. Let λ denote the synthetic PE sn2/sn1 ratio and let $\lambda_{\text{min}} = 2.27$, $\lambda_{\text{max}} = 3.77$, and $\lambda_{\text{avg}} = 2.94$ denote the min, max, and the average of the λ values respectively. Let $\alpha - \beta$, $\beta - \alpha$ be an observed regioisomeric species (e.g., 18:0-18:2 diacyl PE and 18:2-18:0 diacyl PE in 6 month old mouse heart) and let $\mu = \frac{[\beta]}{[\alpha]}$ be the ratio of the measured concentrations of the fatty acids α and β in the regioisomers. Then

$$[\beta] = \lambda[\alpha - \beta] + [\beta - \alpha]$$
$$[\alpha] = [\alpha - \beta] + \lambda[\beta - \alpha]$$

On the other hand, since the (normalized) concentration of the regioisomers sum to 1, we get

$$[\alpha - \beta] = \frac{-(1 - \mu\lambda)}{(\lambda - \mu) - (1 - \mu\lambda)}$$
$$[\beta - \alpha] = \frac{(\lambda - \mu)}{(\lambda - \mu) - (1 - \mu\lambda)}$$

Note that in the above equations, if $\lambda < \mu$ we get that $[\alpha - \beta] > 1$ and $[\beta - \alpha] < 0$. Hence we need to correct the above equation by applying the additional constraint that for $\lambda < \mu$, we set $[\alpha - \beta] = 1$ and $[\beta - \alpha] = 0$, i.e., only one isomer will be assigned. This will also be the case for $\lambda = \mu$. Figure 2 shows the $[\alpha - \beta]$ surface.

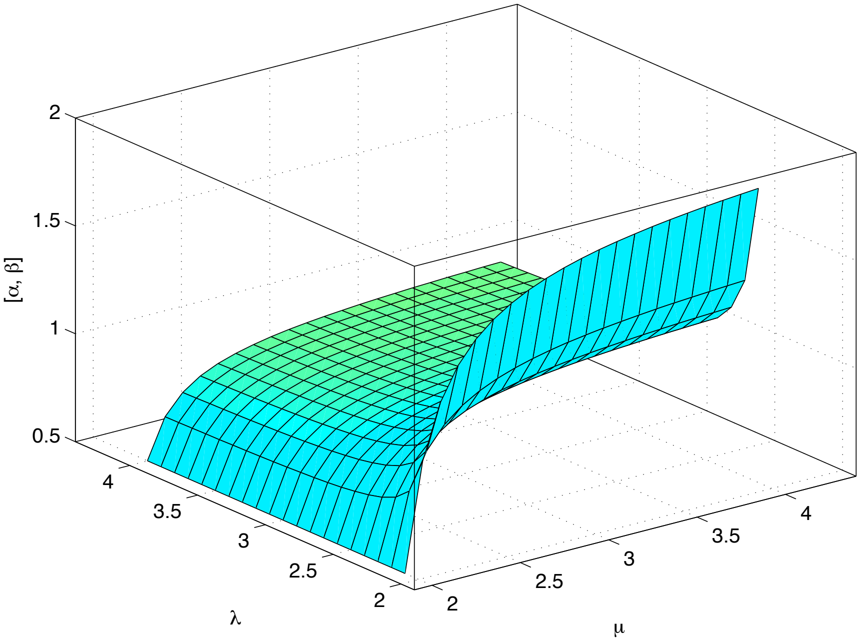

Figure 2: Concentration of the $[\alpha, \beta]$ **isomer.** For a fixed observed μ value, the concentration of the $[\alpha, \beta]$ decreases with increasing values of λ.

As can be seen, the concentration of each regioisomer depends on the choice of λ. However, the λ dependence on regioisomer concentrations is rather weak in the regime $\lambda \in [2.27, 3.77]$, the range of λ values observed experimentally. In the analysis performed in this work, λ was set 3 (roughly the mean value) for all the samples.

To see how the choice of λ affects the subsequent statistical analysis, we first reperformed the clustering analysis on PE data for 6 month old mouse heart for $\lambda = \lambda_{\text{avg}}$, $\lambda = \lambda_{\text{min}}$, $\lambda = \lambda_{\text{min}}$. The experimentally observed ratios of fatty acid chains from carboxylate ions are shown in Table 1. Figure 3 shows the result of the clustering analysis for different values of λ. The left column shows the conditional chain distributions given the sn1 chain type. As can be seen the effect of λ on the clustered chain types is minimal for λ_{min}, $\lambda = \lambda_{avg}$, and $\lambda = \lambda_{\text{max}}$. Here the only noticable effect is that 22:5 and 20:4 are not observed at the sn1 position for the λ_{min} case, but these species are rare at the sn1 position in all three cases, as can be seen in the right column. Similarly, in the right column the only notable differences occur when the the sn2 chain is 16:0, 18:1, or 22:5. But these chains are all rare at the sn2 position.

Table 1: Major PE species in 6-mon mouse heart

Species	Isobaric Ratio	Content (nmol/mg protein)	Acyl Chain Ratio
16:0-18:2/16:1-18:1	10:1	0.19	2.70/1.60
16:0-18:1		0.10	1.20
16:1-20:4		0.07	1.85
16:0-20:4/18:2-18:2	5:1	0.34	4.00/ 1
18:1-18:2		0.22	3.90
18:0-18:2/18:1-18:1	10:1	0.82	6.20/1
18:0-18:1		0.26	3.00
18:0-18:0		0.88	1
16:1-22:6		0.30	5.70
16:0-22:6		7.59	3.37
18:1-20:4		1.23	3.90
18:0-20:4		4.40	2.83
18:2-22:6		1.32	2.89
18:1-22:6		4.79	3.37
18:0-22:6		20.68	1.76
18:0-22:5		1.55	2.29

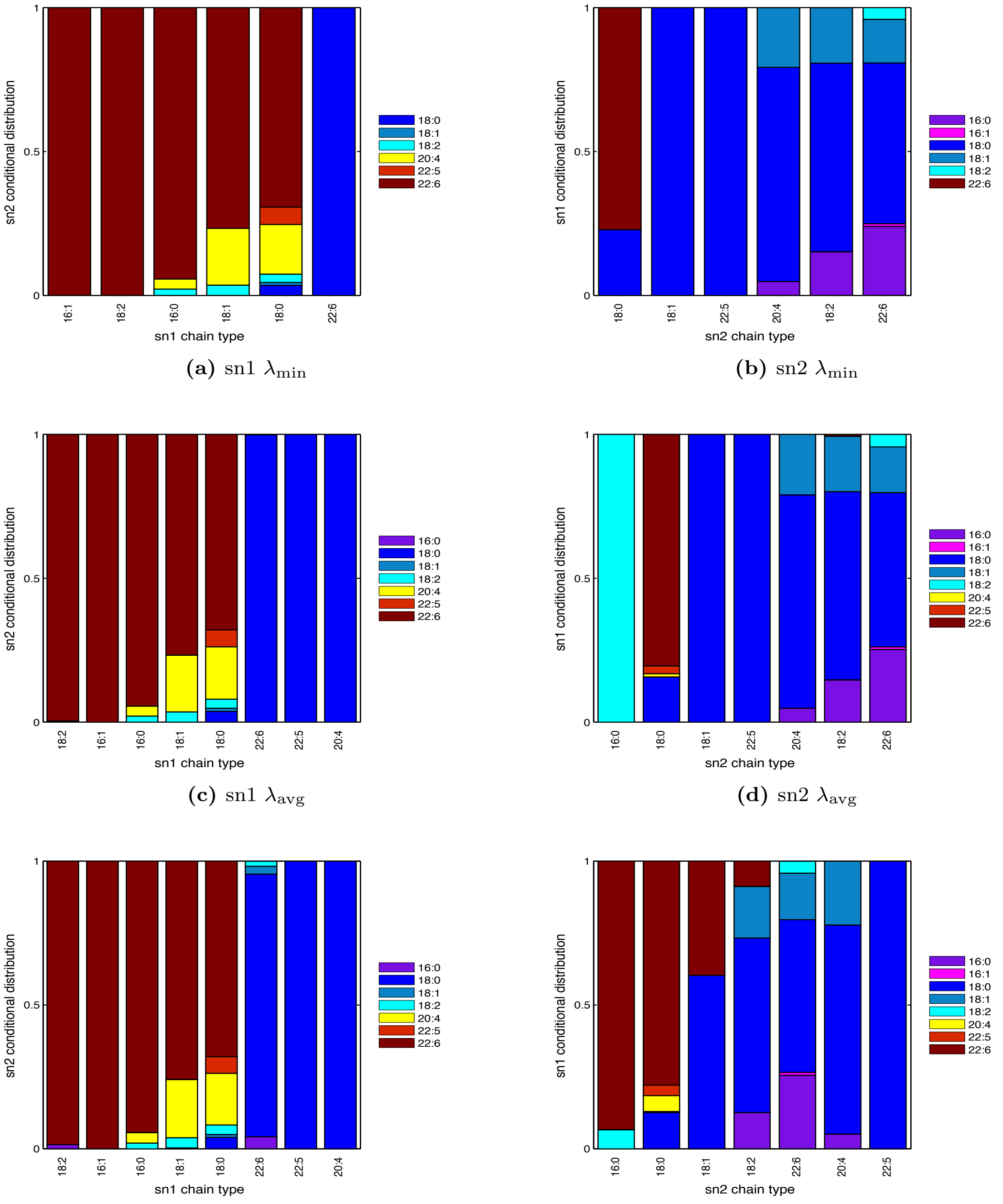

(a) sn1 $\lambda_{\min}$

(b) sn2 $\lambda_{\min}$

(c) sn1 λ_{avg}

(d) sn2 λ_{avg}

(e) sn1 $\lambda_{\max}$

(f) sn2 $\lambda_{\max}$

Figure 3: Clustering of PE for alternative regioisomer assignments. sn1 chain type clustering ($l$eft) and the subsequent sn2 clustering ($r$ight) for $\lambda = \lambda_{\min}, \lambda_{\text{avg}}$, and $\lambda_{\max}$

We next reperformed the test of quasi-independence using the three λ values. As described in the main manuscript, we selected the sn1 and sn2 chain types for testing based on the clustering of behaviors shown in Figure 3 . The results for the largest set of independent chains are shown in Table 2. For all values of λ, the same set of chain types were found in this procedure, and these passed the test of quasi independence at the significance level $p \geq 0.05$. Note that the observed set of chains in the sn1 and sn2 positions are similar to those we had most commonly observed for PE when analyzing the full complement of tissues (Table 3 of the main manuscript). These also tend to be the chain types with high concentrations.

For minor species, the effect of λ may contribute to their apparent deviation from the independence model. However, some of these species consistently deviate from independence in one direction across multiple samples (see section 3). Hence, it is possible that their observed deviations are due to systematic biological effects, such as preferential recognition by regulatory enzymes. For major species the effect of λ on assignment of regioisomer is negligible and does not affect the test of independence.

Table 2: Test of quasi-independence on PE molecular species for alternative regioisomer assignments.

λ	Sample Size	sn1 FA	sn2 FA	p-value
$\lambda_{\text{min}} = 2.27$	1000	18:0, 18:1	18:2, 20:4, 22:6	0.5319
$\lambda_{\text{avg}} = 2.94$	1000	18:0, 18:1	18:2, 20:4, 22:6	0.4732
$\lambda_{\text{max}} = 3.77$	1000	18:0, 18:1	18:2, 20:4, 22:6	0.4645

For each λ, we identified subsets of sn1 and sn2 fatty acids (FA) which passed the independence test ($p \geq 0.05$).

2 Variance effective sample size

In order to estimate the variance effective sample size, we regard each peak k, in the MS data as an independent observation of a binomial process corresponding to the observed species k. Note that k corresponds to the species ij in the Methods. Given L biological replicates, let the effective sample size and the counts of the corresponding specie for the kth peak and the ℓth replicate be n_k and $m_{k\ell}$ respectively. The estimated probability of the kth species in the ℓth replicate is then $\pi_{k\ell} = \frac{m_{k\ell}}{n_k}$. Let

$$\hat{\pi}_k = \frac{1}{L}\sum_{\ell=1}^{L}\pi_{k\ell} = \frac{1}{L}\sum_{\ell=1}^{L}\frac{m_{k\ell}}{n_k} = \frac{1}{n_k}\left(\frac{1}{L}\sum_{\ell=1}^{L}m_{k\ell}\right) = \frac{m_k}{n_k}$$

be the average probability of of the kth species across the replicates. Here m_k is the average count of the kth species. Let s_k be the standard deviation of the counts. For the binomial model $s_k = \sqrt{n_k\hat{\pi}_k(1-\hat{\pi}_k)}$. On the other hand, if $\hat{\sigma}_k$ denotes the sample standard deviation of the probabilities, then

$$\hat{\sigma}_k = \sqrt{\frac{1}{L}\sum_{\ell=1}^{L}(\pi_{k\ell}-\hat{\pi}_k)^2} = \frac{1}{n_k}\sqrt{\frac{1}{L}\sum_{\ell=1}^{L}(m_{k\ell}-m_k)^2} = \frac{s_k}{n_k}$$

From above equation it follows that

$$n_k = \frac{\hat{\pi}_k(1-\hat{\pi}_k)}{\hat{\sigma}_k^2}$$

We then averaged these values of n_k over all k to get an effective sample size n for the tissue type. For this averaging, outlier values of n_k were excluded at the significance level of 0.1, based on an assumed normal distribution for the n_k values.

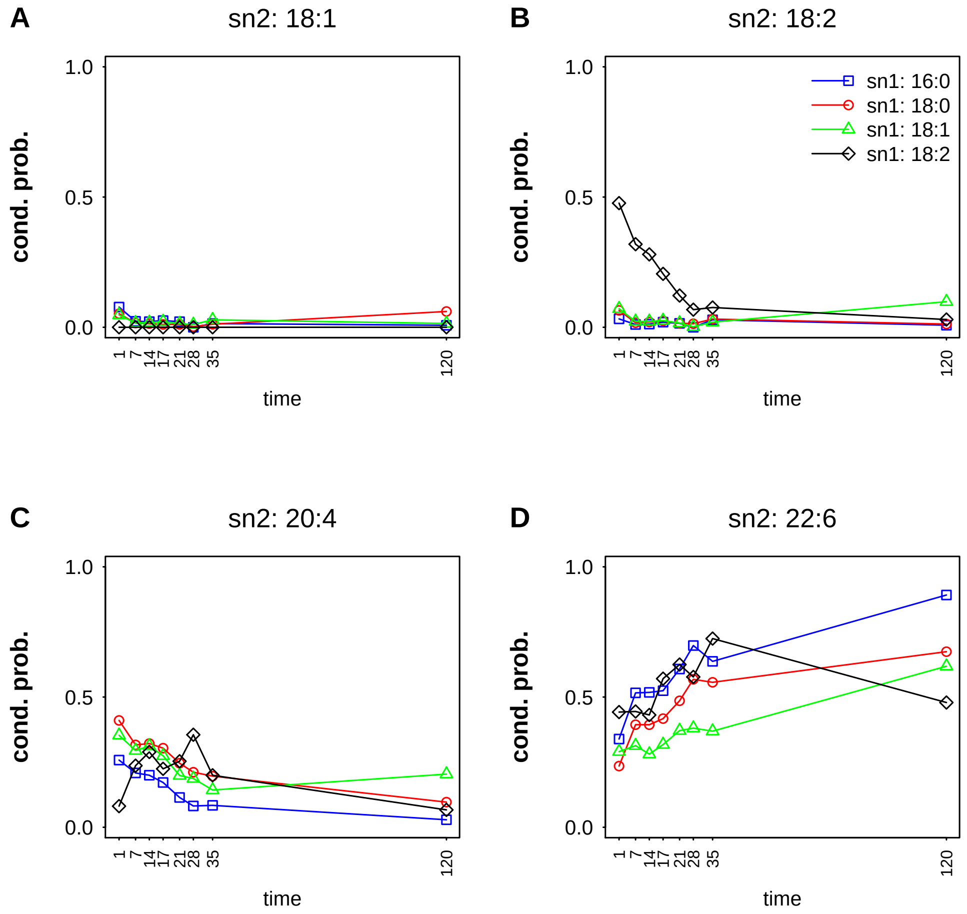

Figure 4: Conditional probabilities of PE during heart development.

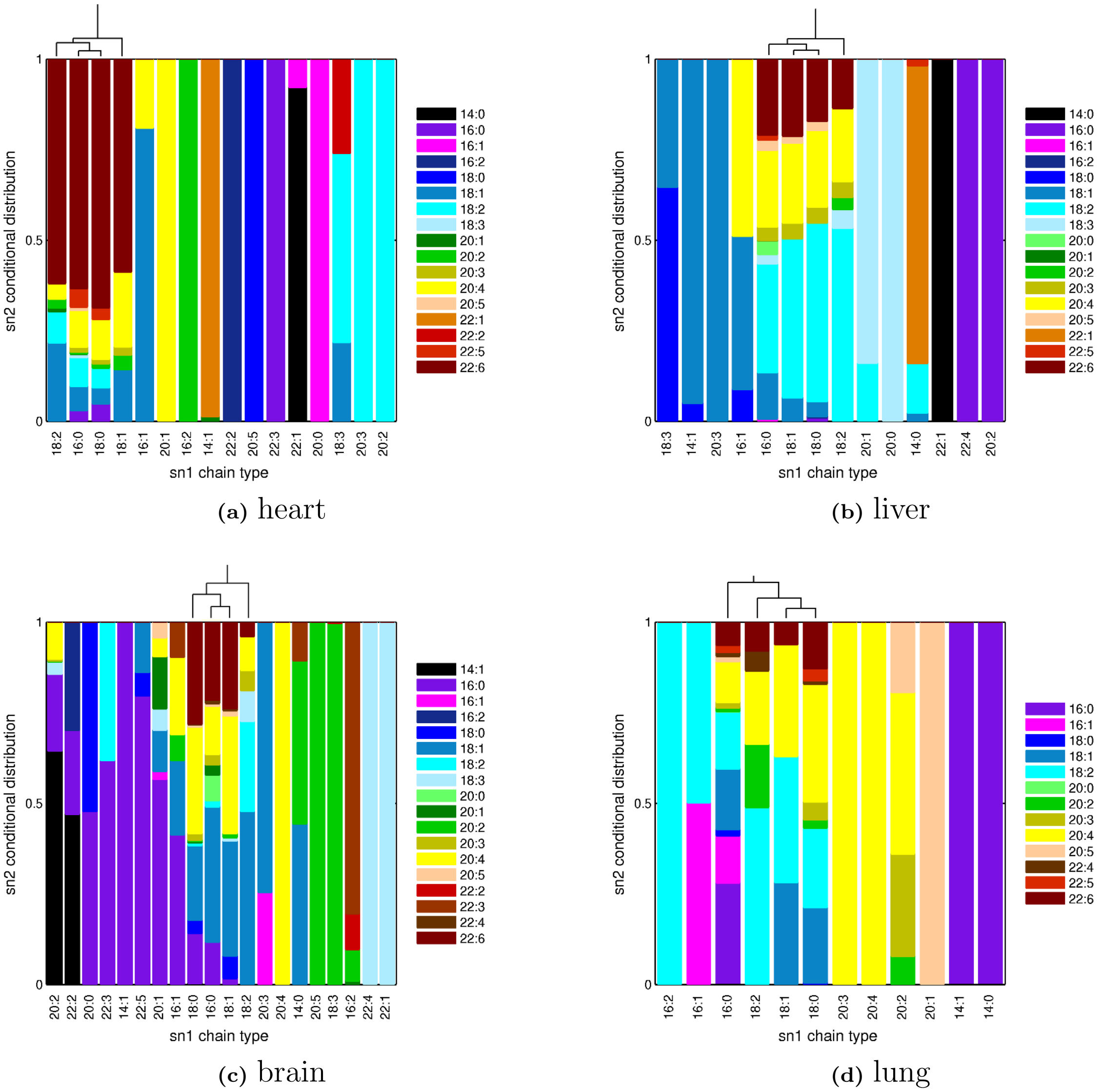

(a) heart **(b)** liver **(c)** brain **(d)** lung

Figure 5: Clustering of PC sn1 chain types.

3 Determination of species causing deviations from the independence model

We performed the following enrichment analysis to test whether the the species that deviate from independence are consistent across samples. Note that the acyl chain ratios used in assigning the regioisomes are specific to the tissue type, but identical across time points. Hence, to avoid bios in the enrichment analysis only mature tissue types are considered.

To determine the species subject to cooperative effects, we calculated standardized residual scores (see Methods). Out of the 394 summed species in all mature mouse tissues, the 39 (10%) with largest absolute deviations were selected, summarized in Supplementary Table 4. All of them have positive deviations, indicating that the cooperative effects may be largely due to synthesis and remodeling rather than degradation. We refer to these 39 species as "deviated species."

Given a species S, we investigated its statistical significance and consistency of deviation across tissue types. We considered a null model where the deviated species are selected at random from the summed sets of species in all tissues. This null tests the hypothesis that a species's probability of being deviated is independent of its particular acyl chains and the tissue type. Under this null hypothesis, the number of times we expect to see a species S as deviated follows the hypergeometric distribution. The statistical significance p-value of species S drawn at least x times randomly, under the hypergeometric distributions was calculated as:

$$p = \sum_{i=x}^{N} \frac{\binom{K}{i}\binom{M-K}{N-i}}{\binom{M}{N}}$$

where M=394 is the total number of observed species summed across all tissue sets; N=39 is the total number of deviated species; and K is the number of tissue types in which species S was found (though not necessarily as a deviated species). See scheme in Supplementary Table 3. At significance level 0.05, we identified 6 species consistently deviating from independence across tissue types, designated by * in Supplementary Table 4, with p-value given in parentheses.

Table 3: Scheme for the analysis of deviated species across tissues.

	heart PC	brain PC	⋯	heart PE	brain PE	⋯	K
Species							
16:0-18:2	3.8095	0.7680	⋯	-4.4039	n/a	⋯	7
16:0-20:4	-0.2603	-3.9704	⋯	-9.7514	-15.9078	⋯	8
16:2-20:2	66.5453^a	6.5804	⋯	n/a	160.6867^a	⋯	3
18:0-20:4	2.5185	9.2460	⋯	-3.1059	0.7850	⋯	8
20:0-16:1	141.5733^a	n/a	⋯	n/a	n/a	⋯	2
20:5-18:0	155.1780^a	n/a	⋯	56.4664^a	132.8561^a	⋯	3
⋮							

a indicates deviated species; K is the number of samples in which the species was measured.

Table 4: Deviated species.

Species	Sample	Std. Residual
14:0-16:0	lung PC	49.7703
14:0-22:1	liver PC	67.9555
14:0-22:2	heart PE	84.8698
14:1-22:1	heart PC	153.8916
16:0-22:6	liver PE	51.3924
16:2-20:2 * (0.0269)	heart PC	66.5453
	brain PE	160.6867
16:2-22:3	brain PC	56.2012
18:2-20:2	lung PC	46.0656
18:3-18:0 * (0.0096)	liver PC	52.294
	liver PE	114.8169
18:3-20:2	brain PC	50.8728
18:3-22:2	heart PC	79.2466
20:0-16:1 * (0.0096)	heart PC	141.5733
	liver PE	124.0923
20:0-20:5	brain PE	153.9105
20:1-16:0	brain PE	49.7449
20:1-18:3	liver PE	43.8106
20:1-20:5	lung PC	60.9589
20:2-14:1	brain PC	45.8118
20:3-16:0	heart PE	57.9983
20:3-20:0	brain PE	161.9745
20:3-20:2	liver PE	54.2487
20:4-16:0	liver PE	72.1205
20:4-20:1	brain PE	120.3832
20:5-18:0 * (9.03e-04)	heart PC	155.178
	heart PE	56.4664
	brain PE	132.8561
22:1-14:0 * (0.0096)	heart PC	149.1648
	liver PC	77.1567
22:2-16:2	heart PC	154.6517
22:3-16:0	brain PE	125.8968
22:4-16:0	brain PE	69.2278
22:4-18:0	heart PE	52.088
22:5-14:1	brain PE	161.6675
22:5-16:0	liver PE	101.6345
22:5-18:0	liver PE	83.727
22:6-18:1 * (0.0096)	brain PE	70.071
	liver PE	120.9603
